# Artificial Neural Network Approach for Modelling of Mercury Ions Removal from Water Using Functionalized CNTs with Deep Eutectic Solvent

**DOI:** 10.3390/ijms20174206

**Published:** 2019-08-28

**Authors:** Seef Saadi Fiyadh, Mohamed Khalid AlOmar, Wan Zurina Binti Jaafar, Mohammed Abdulhakim AlSaadi, Sabah Saadi Fayaed, Suhana Binti Koting, Sai Hin Lai, Ming Fai Chow, Ali Najah Ahmed, Ahmed El-Shafie

**Affiliations:** 1 Nanotechnology & Catalysis Research Centre (NANOCAT), IPS Building, University of Malaya, Kuala Lumpur 50603, Malaysia; 2 Department of Civil Engineering, Al-Maaref University College, Ramadi 31001, Iraq; 3 Department of Civil Engineering, University of Malaya, Kuala Lumpur 50603, Malaysia; 4 National Chair of Materials Science and Metallurgy, University of Nizwz, Sultanate of Oman, Nizwa 616, Oman; 5 Institute of Energy Infrastructure (IEI), Department of Civil Engineering, Universiti Tenaga Nasional, 43000 Kajang, Selangor, Malaysia

**Keywords:** adsorption, environmental modelling, mercury ions removal, deep eutectic solvents, carbon nanotubes, artificial neural network

## Abstract

Multi-walled carbon nanotubes (CNTs) functionalized with a deep eutectic solvent (DES) were utilized to remove mercury ions from water. An artificial neural network (ANN) technique was used for modelling the functionalized CNTs adsorption capacity. The amount of adsorbent dosage, contact time, mercury ions concentration and pH were varied, and the effect of parameters on the functionalized CNT adsorption capacity is observed. The (NARX) network, (FFNN) network and layer recurrent (LR) neural network were used. The model performance was compared using different indicators, including the root mean square error (RMSE), relative root mean square error (RRMSE), mean absolute percentage error (MAPE), mean square error (MSE), correlation coefficient (*R*^2^) and relative error (RE). Three kinetic models were applied to the experimental and predicted data; the pseudo second-order model was the best at describing the data. The maximum RE, *R*^2^ and MSE were 9.79%, 0.9701 and 1.15 × 10^−3^, respectively, for the NARX model; 15.02%, 0.9304 and 2.2 × 10^−3^ for the LR model; and 16.4%, 0.9313 and 2.27 × 10^−3^ for the FFNN model. The NARX model accurately predicted the adsorption capacity with better performance than the FFNN and LR models.

## 1. Introduction

Mercury is the most toxic heavy metal, and has a serious influence on the environment and human health [1]. Mercury poisoning effects mostly include renal disorders and neurological disorders; mercury easily passes through the brain barrier and influences the brain. High mercury concentrations are a source of impaired kidney and pulmonary function [2]. Mercury availability and toxicity depend on the chemical form in which the mercury is found. Mercury can be released from different sources due to its long range, bio-accumulative properties, and high toxicity [3]. The removal of mercury is a substantial concern [4,5]. Elemental mercury (Hg^0^) is highly insoluble and volatile in water; therefore, Hg^0^ removal using traditional methods is problematic [6]. Major problems due to mercury (Hg^2+^) pollution exist in several countries, including Iraq, China, Brazil and Japan [7,8]. The pollution of water by mercury is one of the main health problems for the public, even with low concentrations. World Health Organization (WHO) has determined that the maximum allowable mercury concentration in the drinking water is 2.0 mg/L [9].

However, various techniques have been utilized for mercury ions removal from water, including membrane separation [10], flotation, reverse osmosis [11], precipitation [12], mechanical filtration [13], exchange of ion [14], and adsorption [15]. These traditional methods have some drawbacks; therefore, better methods with high efficiency are required. Adsorption has been proven to be an efficient and cost-effective method, and it is strongly recommended [16]. The effectiveness of adsorption is primarily dependent on the selection of appropriate process conditions, including the mass of sorbent, pH, system temperature and process duration [17]. Many adsorbents have been used for heavy metal removal, including activated carbon, magnetic adsorbents [18], low-cost natural materials [19], biomaterials, clay minerals [20] and carbon nanotubes (CNTs) [21].

A highly efficient adsorption material with a high sensitivity and surface area for Hg^2+^ detection and absorption is required. CNTs have different physicochemical properties from those of other materials used as adsorbents; these different properties make CNTs suitable for many applications in water treatment, nanotechnology, electronics, optics and other material science fields [22,23]. Regardless of the limitations of CNTs, such as difficulty in manipulation, aggregation and solubility, CNTs have excellent properties due to interactions with other compounds by surface modification [24,25].

Lately, there has been growing interest in the development of green and environmentally friendly solvents through analytical studies. Deep eutectic solvents (DESs) have attracted great interest as green solvents in many applications [26]. DESs have great benefits in terms of ease of synthesis, low toxicity and availability of material. DESs are analogous to ionic liquids (ILs); DESs are used as cheap IL replacement materials and are environmentally friendly [27]. DESs were presented by Abbot et al. in 2003 [28]. DESs consist of two or more safe components that can associate with each other by mostly occasional hydrogen bonding, electrostatic interactions and van der Waals forces [29,30]. The DES melting point is lower than the melting points of individual components.

The adsorption process proposed in this study is considered an effective process compared to other techniques; however, it is a complicated process, because there are many influential variables, including pH, contact time, mercury concentration and adsorbent dosage, and it has not yet been standardized and modelled. Consequently, a powerful modelling technique is required. ANNs are a powerful technique that is able to categorize given data to its outputs. The ability of ANNs to generalize and learn complex and non-linear process behaviours makes them a powerful method [31]. Recently, ANN techniques have been used for several engineering applications [32,33,34].

In previous research, the authors used the tetra-n-butyl ammonium bromide (TAB) and glycerol (Gly) to prepare the DES as a functionalization agent of CNTs [35]. Meanwhile, in the present research, allyl triphenyl phosphonium bromide (APB) and glycerol (Gly) are mixed to prepare the DES. In fact, the behaviour of these two DESs is completely different in term of CNT functionalization for mercury removal from the water. In this context, it was necessary to investigate the potential of implementing the same modelling method. In addition, due to the experimental constraints, it was necessary to carry out more experimental replicates in the current research (176 samples) than in the previous research (163 samples). On the other hand, the second major difference is the modelling methods used in the current research, which comprised three different algorithms rather than just a single ANN algorithm used in the previous research. This is due to the fact that there is a need to examine different modelling methods to achieve an accurate prediction for the removal of mercury from the water.

The objectives of this work are to use a DES comprising a combination of allyl triphenyl phosphonium bromide (APB) and glycerol (Gly) to functionalize CNTs; to use the DES-functionalized CNTs as an adsorbent for mercury ions removal from water solution; to use the NARX, FFNN and LR neural network approaches to model the adsorption process; and to compare the model behaviour in terms of accuracy and productivity.

## 2. Results and Discussion

The removal of mercury from water solution and the neural networks used as a modelling technique for modelling the functionalized CNT adsorption capacity were the main focus of this study. Ak-CNTs were used in this study, and different amounts of the adsorbent were used—5, 20 and 30 mg. The efficiency of the adsorbent was examined using various initial concentrations—1, 3 and 5 mg/L—and different pH values—3, 5.5, 6 and 8. The contact time lasted until equilibrium was reached. Different samples were used in experiments at different times using various variable values, including mercury concentration, adsorbent dosage and pH; therefore, the total number of samples prepared was 176. The effect of the involved input parameters on the adsorbent (Ak-CNTs) adsorption capacity is studied. The evaluation of model performance was conducted using different indicators, including RRMSE, MSE, RE, RMSE, and MAPE.

### 2.1. Hybrid Material Characterization 

Investigating the electrical charge of adsorbent is important because of the parameter effect on the adsorbent’s efficiency. The measurement of zeta potential electrical potential on the dielectric layer on the suspended particles surface in the bulk fluid and solution. The electric potential is the balance of the electrostatic force which keep the microparticles or nanoparticles stable in a suspension or emulsion. The zeta potential measurement for the P-CNTs and Ak-CNTs are conducted, the zeta potential absolute value increased, the P-CNT value is 5.5 mV, whereby the value of the Ak-CNTs is 52.3 mV. Furthermore, an increase in the ID/IG ratio was presented using the Raman spectra from 1.11 to 1.18 for the P-CNTs and Ak-CNTs, respectively. This displays the functional groups effect in the sp3 direction. Furthermore, Raman spectra presented an increase in the ratio of ID/IG from 1.11 to 1.18 for the P-CNTs and Ak-CNTs, respectively, which displays the functional groups effect in the sp3 direction gained from A-DES. The results showed that O-H stretching disappeared after A-DES functionalization. O-H may have been present because the adsorbed water on the CNT surface was hydrophilic, which was obvious in the k-CNT case. A-DES functionalization enhanced the sample drying process and decreased the hydrophilicity, which is why OH- disappeared from the P-CNT and Ak-CNT FTIR spectra. PO_4_^−3^ is observed in the 500–600 cm^−1^ range, and C-Br stretching was also detected in the 550–650 cm^−1^ range. The adsorption process is influenced by the adsorbent surface area; therefore, the use of A-DES as a CNT functionalization agent increased the pore size diameter of adsorbent from 20.49 to 127.34 Å and the surface area from 123 to 199.366 m^2^/g. The increase resulted in the performance of the adsorption capacity of Ak-CNTs [36].

### 2.2. pH Effect

**Figure 1:**
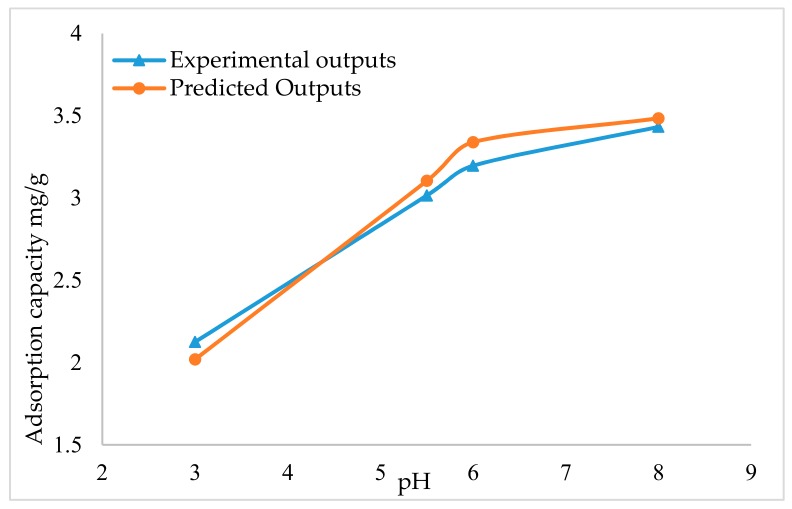
Effect of pH on adsorption capacity.

Solution pH is one of the main operational variables in water treatment systems in industrial, commercial and urban areas. The effect of pH is studied to optimize the value for absorbance or purification. Deviations at this point in experiments and parameter sensitivity analysis will result in high uncertainty and poor performance. The effect of pH on Ak-CNTs is presented in Figure 1. The pH effect is investigated by fixing the other parameters, including an adsorbent dosage of 20 mg, contact time of 55 min, and 3 mg/L initial concentration. The pH values were 3, 5.5, 6 and 8. pH has an effect on functional group protonation in biomass, such as in amino phosphate and carboxyl groups, and the metal chemistry, including the solubility [37,38]. The results reveal that with increasing pH, the adsorption capacity increases as well. Increasing the value of pH from 3 to 5.5, an increase in the adsorption capacity occurred from 2.125 mg/g to 3.015 mg/g, while increasing the value of pH from 6 to 8 increased the adsorption capacity from 3.196 mg/g to 3.432 mg/g. This increase occurred due to the presence of negatively charged functional groups that contain oxygen, such as carboxylic groups, and the enhancement of negative electronic charge by the presence of OH- in the solution. This negative charge pattern was widely distributed on the adsorbent surface, which may determine metal sorption. At pH values greater than 7, the Hg^2+^ dominant species are Hg (OH)^+^ and Hg (OH)_2_. This complexation occurs due to the presence of OH−, which results in precipitation [32]. The NARX network modelling method is used to model the adsorption capacity using the obtained experimental data set. Upon comparing the NARX outputs to experimental results, the NARX model showed high accuracy; Figure 1 presents the NARX and experimental results.

### 2.3. Initial Concentration Effect

**Figure 2:**
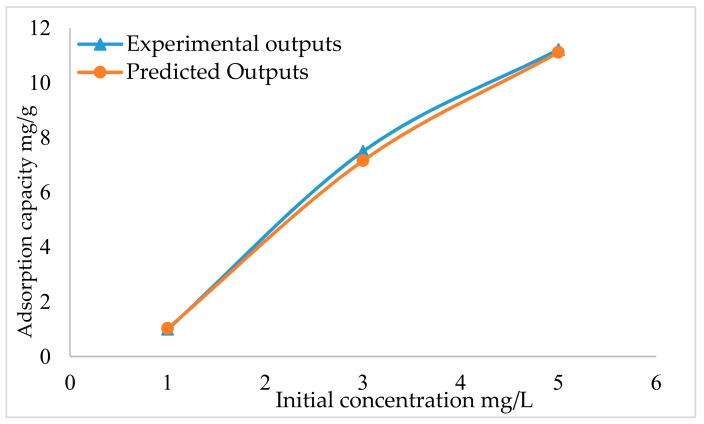
Effect of initial concentration on adsorption capacity.

The initial Hg^2+^ ion concentration is used as one of the parameters in the experimental work, and the Hg^2+^ initial concentrations used were 1, 3 and 5 mg/L. The effect of Hg^2+^ concentration on Ak-CNTs is presented in Figure 2. The Hg^2+^ initial concentration had a favourable and prominent effect on the Hg^2+^ adsorbed quantity on the Ak-CNT adsorbent. 

The effect of the initial concentration of mercury ions on the Ak-CNT adsorption capacity was investigated by fixing a contact time of 120 min, pH of 5.5 and adsorbent (Ak-CNT) dosage of 5 mg. Upon increasing the mercury concentration, the adsorption capacity is improved. The adsorbent (Ak-CNT) adsorption capacity is increased from 0.9935 to 7.495 mg/g by increasing the concentration from 1 to 3 mg/L; meanwhile, when increasing the mercury concentration from 3 to 5 mg/L, the adsorbent (Ak-CNT) adsorption capacity increased from 7.495 to 11.214 mg/g. This increase, caused by the higher collision between the adsorbent molecules and adsorbent (Ak-CNT) active sites [39]. The experimental results were used to train the NARX neural network model, and the created model proved to have high accuracy compared to the experimental data. Figure 2 presents the NARX and experimental results.

### 2.4. Effect of Adsorbent Dosage 

**Figure 3:**
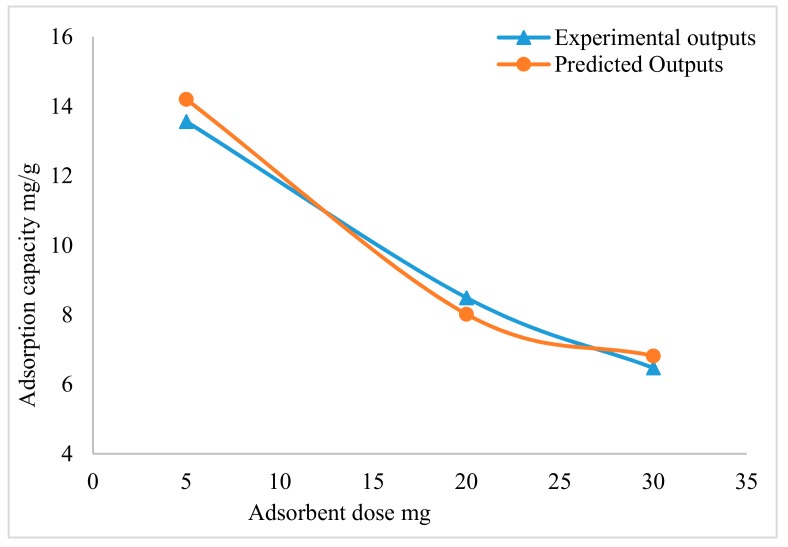
Effect of adsorbent dosage.

The adsorbent dosage effect on the adsorption capacity has an important influence, the Ak-CNTs were used as an adsorbent in this study for mercury removal. The adsorbent dosages used in this study were 5, 20 and 30 mg. To examine the adsorbent dosage effect, the other parameters involved in the experimental work were fixed, with a contact time of 30 min, mercury initial concentration 5 mg/L was selected, and pH value 6. The presented results in Figure 3 reveal that the adsorption capacity decreased when increasing the adsorbent dosage. This decrease might have happened due to the fact that during the adsorption process, some of the active sites remained unsaturated [40,41]. The adsorbent adsorption capacity was 13.56 mg/g when using an adsorbent dosage of 5 mg; meanwhile, by increasing the adsorbent dosage amount from 20 to 30 mg, there was a decrease in the adsorption capacity from 8.49 to 6.47 mg/g. 

The results achieved from the experimental work were used to train the NARX neural network model. The model results are compared to the experimental result, the NARX model proved high accuracy in the predicting the Ak-CNT adsorbent adsorption capacity. The NARX model and experimental results are presented in Figure 3.

### 2.5. Kinetic Study

**Figure 4:**
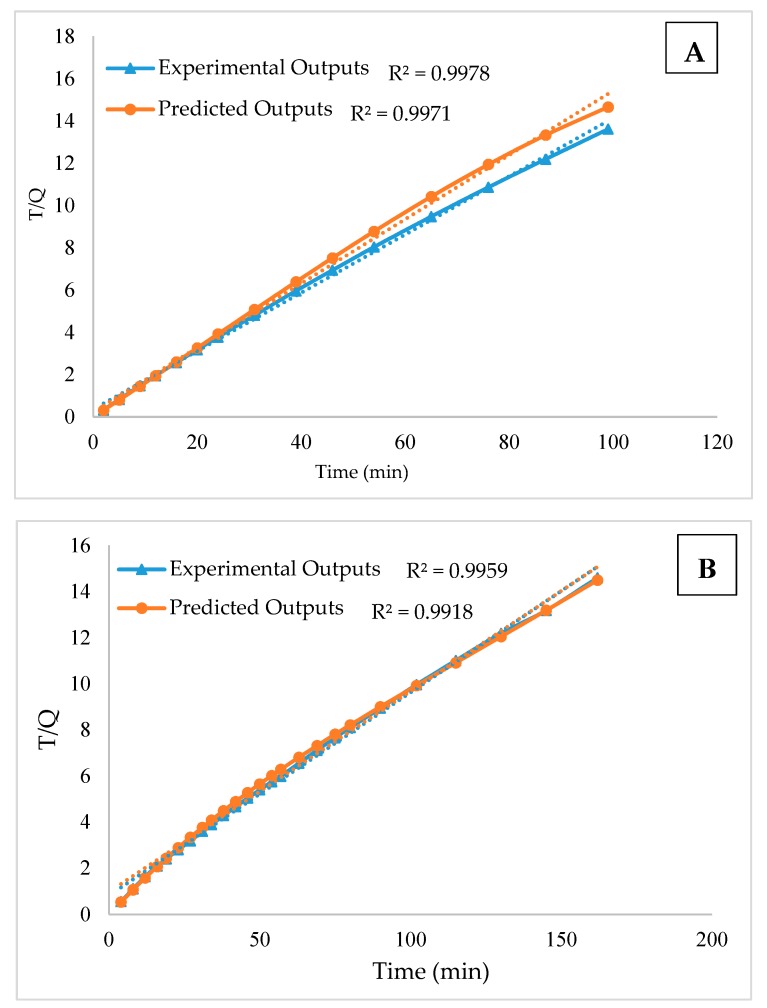

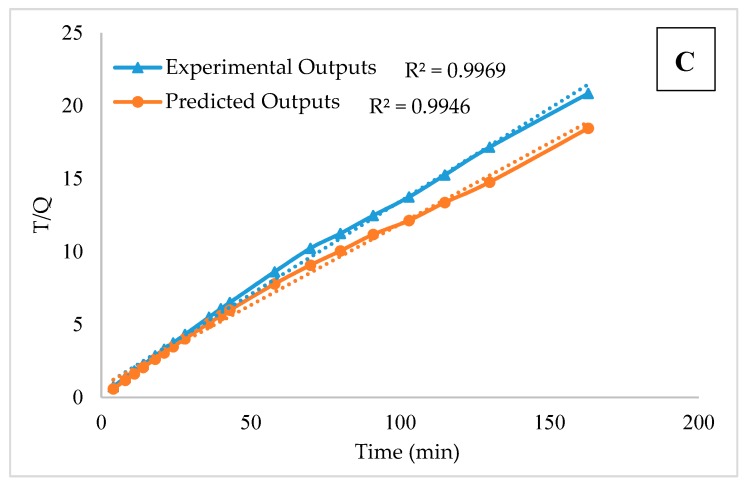
Kinetic analysis for the experiment (**A**) 20 mg of adsorbent dosage, 3 mg/L of initial mercury concentration, pH of 3; (**B**) the adsorbent dosage used is 20 mg, the concentration of mercury ions is 5 mg/L, the selected pH is 6 and (**C**) the adsorption capacity is 30 mg, the initial concentration of mercury ions is 5 mg/L, the used value of pH is 6.

The kinetic study aimed to study the behaviour of the reaction using the Ak-CNT adsorbent. The kinetic models were applied, including pseudo first-order, pseudo second-order and intraparticle diffusion models. Different parameter conditions were used in this work for the kinetic study. Figure 4A presents the kinetic study results using 20 mg of adsorbent dosage, 3 mg/L of initial mercury concentration, pH of 3 and different time intervals until reaching equilibrium. In Figure 4B, the adsorbent dosage used is 20 mg, the concentration of mercury ions is 5 mg/L, the selected pH is 6, and the contact time (time to equilibrium) was until 162 min. In Figure 4C, the adsorption capacity is 30 mg, the initial concentration of mercury ions is 5 mg/L, the used value of pH is 6 and the contact time is 163 min. Figure 4A–C indicates that the Ak-CNT adsorption process fits to a pseudo-second order, with a higher correlation coefficient *R*^2^ value compared to the results of the pseudo first-order and intraparticle diffusion models [42]. Table 1 presents the kinetic models results. The NARX modelling method is used to model and predict the experimental results. The kinetic models were applied on the NARX model outputs, the results also fitted to pseudo second-order. The kinetic study results fit to the pseudo second-order model with *R*^2^ values greater than 0.99 at different doses, initial concentrations and pH values. The NARX output result was also fitted to the pseudo second-order model with an *R*^2^ value greater than 0.99 at the same dose, initial concentration and pH, confirming that the NARX model is able to predict the experimental results. The kinetic model results are presented in Table 1.

### 2.6. Neural Network Performance

The removal of mercury from water by Ak-CNT adsorbent was modelled by ANNs using MATLAB R2014a software. The experimental data set prepared at the lab scale with a total of 176 data points which is used to model the adsorption capacity of Ak-CNT adsorbent. The contact time, initial concentration, pH and adsorbent dosage are included in the experimental work; later, the used parameters were included in the modelling process, the used parameters values were: adsorbent dosage amount (5, 20 and 30 mg), concentration of mercury ion (1, 3 and 5 mg/L), contact time (varying intervals), and pH (3, 5.5, 6 and 8). One hundred seventy-six (176) datasets were prepared at the lab scale. One hundred fifty-one (151) records were used for the network training and validation and twenty-five (25) records were used for testing.

**Figure 5:**
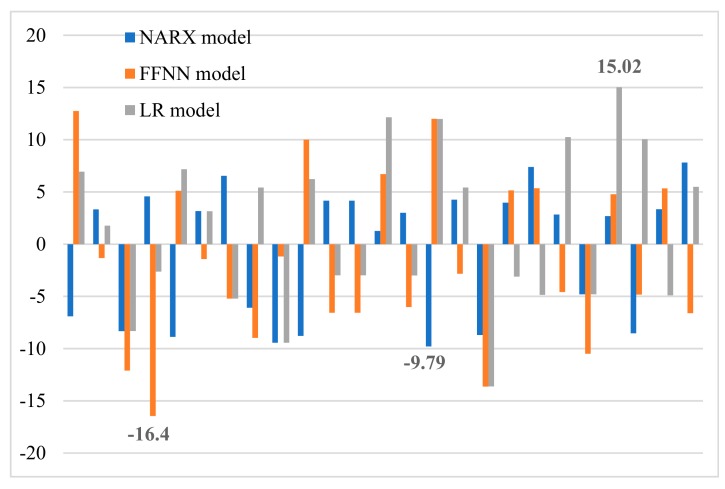
Relative error (RE) of the NARX model.

**Figure 6:**
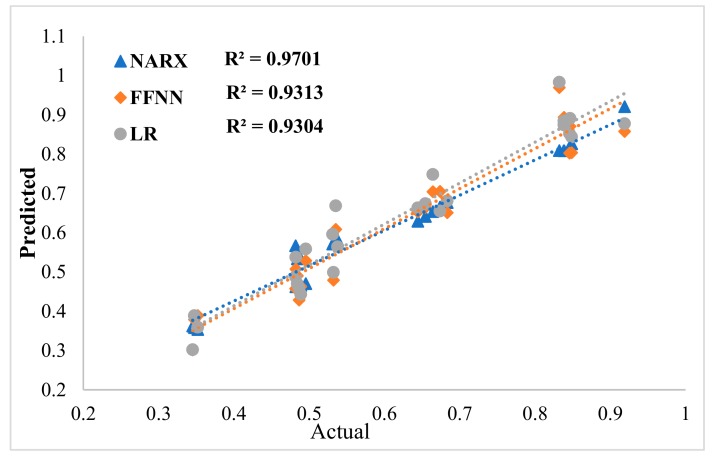
Coefficients of correlation (*R*^2^) of the neural network models.

In this work, three neural network types were used and compared based on their productivity and performance. The best model was used to study the parameters effect including pH, adsorbent dosage, and initial concentration and for kinetic studies. The NARX model has a maximum RE of 9.79% (Figure 5) and *R*^2^ of 0.9701 (Figure 6). In contrast, the FFNN model has a maximum RE of 16.4% (Figure 5) and *R*^2^ of 0.9313 (Figure 6). The LR model has a maximum RE of 15.02% (Figure 5) and *R*^2^ of 0.9304 (Figure 6).

Comparison of the RE and *R*^2^ values for the three models reveals that the NARX model has the best performance. The selection of a proper model structure with high accuracy and the productivity is a challenging task because of the involvement of different parameters, such as the hidden layer(s) number, transfer function type and the neuron(s) number in each layer. In this study, different hidden layer numbers, transfer function types and node numbers were used to choose the suitable structure. The structure selection was based on the performance and the productivity of the network. Initially, the MSE was used during the training phase; then, the created model performance and productivity were tested based on data that were not included in the training section using various indicators; the results are presented in Table 2. By comparing the obtained results, which are presented in Table 2 for the three models, the NARX model showed better performance than the FFNN and LR models. 

## 3. Materials and Methods

### 3.1. Chemicals and Experiments Setup 

The chemicals and materials utilized in this work included multi-walled CNTs with dimensions (D × L) of 6–9 nm × 5 µm and a content of >95% carbon, hydrochloric acid (36.5%–38%), sodium hydroxide pellets, Gly and potassium permanganate, all of the materials were provided by SIGMA-ALDRICH. APB and a 1000 mg/L mercury standard solution were provided by MERCK.

The DES synthesis was conducted by mixing the Gly with APB using the magnetic stirring system at 400 rpm with a temperature of 80 °C. The mixing continued until the mixed component reached to the liquid state without precipitation; the product is referred to as A-DES in this study. The molar ratio details, synthesis and characterization were based on [43]. Primary oxidation was conducted to oxidize the pristine CNT (P-CNT) surface with KMnO4 for 2 h at 65 °C [44]; the CNTs functionalized with KMnO_4_ (adsorbent) are symbolized as k-CNTs. Then, 200 mg of k-CNTs was mixed with 7 mL of the prepared A-DES using the sonication system for 3 h at a temperature of 65 °C to produce Ak-CNTs as an adsorbent. After the functionalization steps, a washing step was conducted using distilled water with a vacuum pump and a polytetrafluoroethylene (PTFE) 0.45 µm membrane until the filtered water pH became neutral. Then, the adsorbent (Ak-CNT) was dried overnight at a temperature of 100 °C. 

The adsorption process was conducted with different values for the involved parameters, including pH, initial mercury concentration, contact time and amount of adsorbent dosage. The Ak-CNT adsorbent was prepared to remove mercury ions from water solution; three different amounts of Ak-CNTs were used (10, 20 and 30 mg), the utilized mercury ion concentrations were (1, 3 and 5 mg/L), the values of pH were 3, 5.5, 6 and 8 and the contact time lasted until equilibrium was achieved. Different amounts of Ak-CNTs were mixed with 50 mL of Hg^2+^ stock solutions in 250 mL flasks with different pH values and mercury concentrations. The prepared flasks were placed in the mechanical system with a 180 rpm shaking speed at room temperature. 176 samples were used in this work under various conditions. The samples were tested at different time intervals to study the adsorbent performance. Inductively coupled plasma-optical emission spectrometry (ICP-OES) (OPTIMA7000DV, PerkinElmer^®^, USA) was used to test the samples.

Zeta potential analysis, Raman spectroscopy and Fourier transform infrared (FTIR) were utilized to characterize the Ak-CNTs, k-CNTs and P-CNTs. To identify the surface charge of the adsorbent particles, the Zetasizer (Malvern, UK) was used. To determine the surface chemical modification of the adsorbents, a PerkinElmer^®^ FTIR spectrometer (Akron, OH, USA) with a wavenumber range of 400–4000 was used. The Raman shift was obtained using a Renishaw System 2000 to identify the functionalization degree.

### 3.2. Artificial Neural Networks (ANNs) 

ANNs are formed from single neurons that are connected and arranged in a way that is described by their architecture [45,46]. There are three stages of modelling: training, validation and testing. During the training stage, a dataset is fed to the network, and a selected algorithm works to fit the provided dataset by adjusting the weights that connect the neurons. During the training stage, transfer functions, such as sigmoid or linear functions, determine the calculations that occur during the processing of the data. Nonlinear responses between the classification and sigmoid transfer functions enable the ANN to detect nonlinear relationships during training of the dataset [47]. ANNs are a mathematical method based on biological neural systems and have the ability to learn, store and recall information. The multi-layer perceptron (MLP) neural network is the most common and simple ANN that is allocated to FFNNs. FFNNs consist of multiple layers. The connection arrangements between the layers permit information to pass forward to the output layer only. Some networks use feedback connections that permit information to pass backwards or laterally within the network; these networks are named recurrent neural networks (RNNs) [48]. The NARX network is a nonlinear network with an exogenous input [49]. During pre-processing, the data are normalized between 0 and 1 to avoid network over-fitting. In this work, an FFNN, LR neural network and NARX neural network are proposed to model the functionalized CNTs adsorption capacity.

Moreover, FFNNs have several neurons; the neurons transfer the input value to the next layer. FFNNs use a supervisory learning method to select the optimum parameters, such as the weight and bias value [50]. In FFNNs, the neurons in the same layer do not connect to each other but are rather connected to the next layer. The layer connection is expressed by the weight value [51]. FFNNs use learning to produce the relationship between the inputs and the target data, which is usually associated with a random initial weight, and then update the value by comparing the network results with the experimental results. In the diverse research using neural computations, different transfer functions are used depending on the problem nonlinearity and data complexity to design proper networks. The selection of a proper network structure depends on different variables, such as the type of transfer function, number of hidden layer and number of neurons. The best FFNN model performance was obtained by using three hidden layers and 10 neurons with a tansig as a transfer function.

The LR neural network is a recurrent network, and each layer of the network has a recurrent connection associated with the hidden layer and the output layer. The tap delay associated with the network permits the network to produce a dynamic response to input samples in a time series [52]. Moreover, the LR neural network is similar to the distributed delay network and time delay with a finite impulse response. The LR neural network is classified by the backward connection from the hidden layer output to the hidden layer input as a context unit. The selection of a proper network structure depends on different variables, such as the type of transfer function, hidden layer number, and neurons number in the hidden layer. The best LR neural network model performance was obtained by using two hidden layers and 15 neurons with a tansig transfer function.

NARX is a recurrent neural network. It is a nonlinear network and has an exogenous input. NARX consist from different layers such as input layer, hidden layer and the output layer with a feedback connection [49]. NARX has the highest generalization degree and speed of convergence compared to the other networks. The NARX network uses an iterative training process; weight and biases are iteratively updated to develop the performance of the model at each step. The network outputs are regressed with actual target values during the training step, with the actual data being fed to the network during the training phase. This method results in better training and learning, and good performance of steady networks such as FFNN. 

With regard to the inclusion of an exogenous input, the NARX model has a greater degree of freedom compared with other networks. This degree of freedom decreases the number of parameters required by the model and increases the model accuracy. The NARX outputs are presented in Equation (1).

(1) $$y(t)=f[u(t-n_{u}),\ldots ,u(t-1),\;u(t),\;y(t-n_{y}),\ldots ,y(t-1)]$$

where: *f* = non-linear function, *y*(*t*) = network output, *u*(*t*) = network input, *n_y_* and *n_u_* = output and input order. 

**Figure 7:**
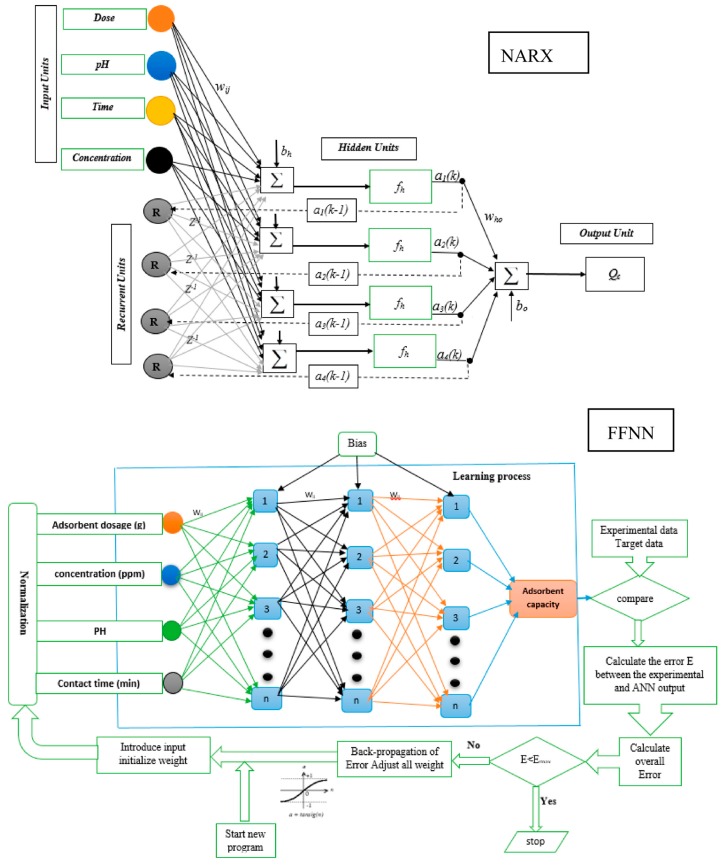

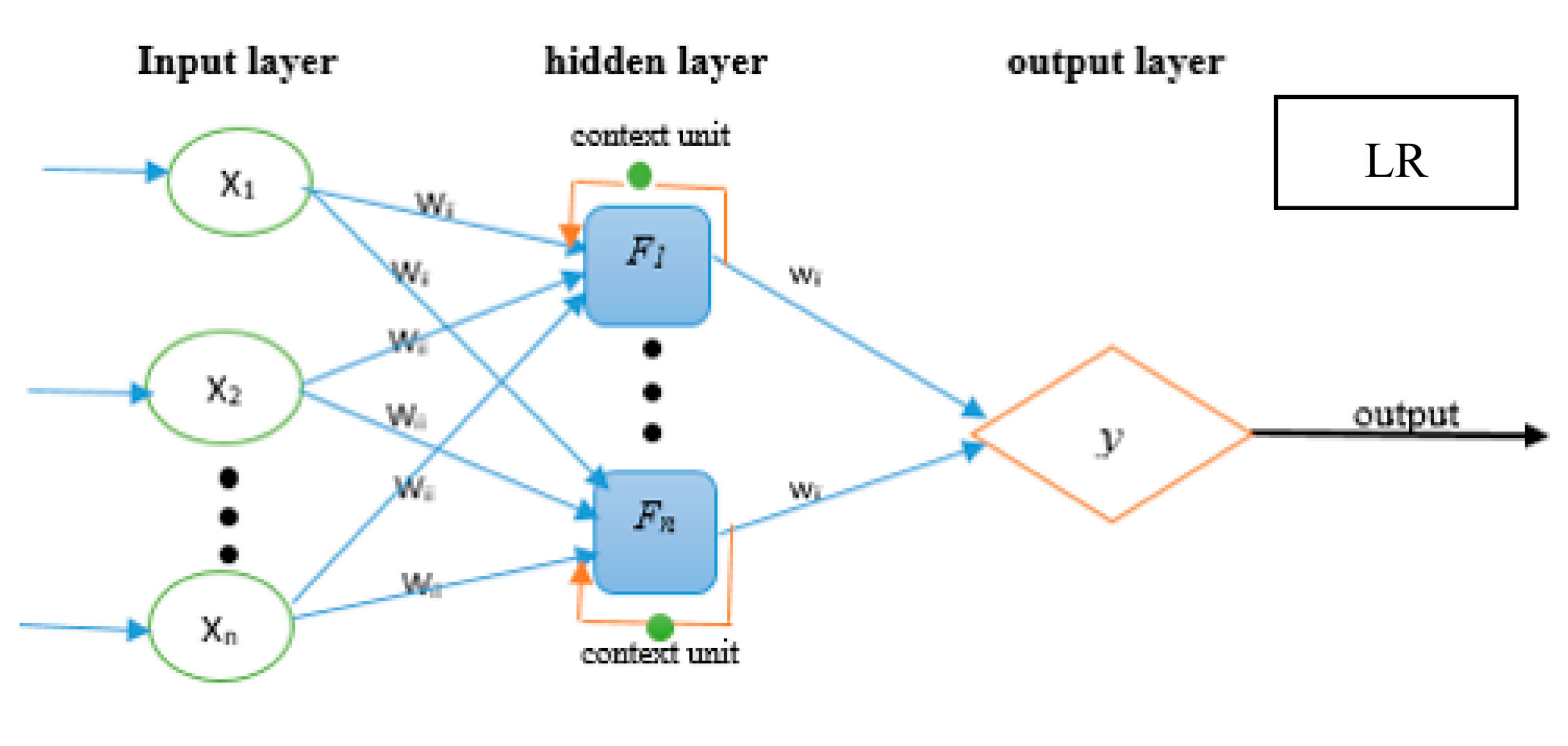
The NARX, FFNN and LR neural network structures.

When *f* constitutes a multi-layer perception, the system result is identified as a NARX network [49]. It is determined that the best NARX structure uses three hidden layers, and the general models’ structures are presented in Figure 7. One input layer is used with four (4) input nodes (initial concentration of mercury ions, pH, adsorbent dosage and contact time) and one output layer with one node (*Q_c_*). In the Figure 7, the *z* is the delay element, *w_ij_* is the weight of network and *b_h_* is the bias of network. The development of the NARX model contains several steps (training and validation) using the same data set and testing for the model accuracy. For the training, validation and testing step, 176 runs were used for the development of the NARX model. The training and validation steps were developed in parallel, pre-switching to the testing step. For training and validation, 151 runs were used, while the remaining 25 data sets were used for the model testing. For the training of the model, the trainbr is the best selection with the best performance. Different network structures were utilized to select the best structure with the lowest error. The selected optimum structure consists of one input layer with 4 nodes, three hidden layers with 10 neurons, and one output layer with one neuron; the best transfer function is tansig, and was used for the modelling. 

Different indicators are selected in this work to evaluate the model’s accuracy and performance using the actual and predicted results. The used indicators are RE, RMSE, MSE, MAPE and RRMSE.

(2) $$RRMSE={\left[\frac{1}{n}\sum_{t=1}^{n}{\left(\frac{D_{a}(t)-D_{f}(t)}{D_{a}(t)}\right)}^{2}\right]}^{\frac{1}{2}}$$

(3) $$MSE=\frac{1}{n}{\sum_{i=1}^{n}\left(D_{a}(t)-D_{f}(t)\right)}^{2}$$

(4) $$RMSE={\left[\frac{1}{n}\sum_{t=1}^{n}{\left(D_{a}(t)-D_{f}(t)\right)}^{2}\right]}^{\frac{1}{2}}$$

(5) $$MAPE=\frac{1}{n}\sum_{t=1}^{n}\left|\frac{\left(D_{a}(t)-D_{f}(t)\right)}{D_{a}(t)}\right|\times 100$$

(6) $$RE=\frac{D_{a}(t)-D_{f}(t)}{D_{a}(t)}\times 100$$

where: $D_{f}(t)$ = the predicted results, $D_{a}(t)$ = the actual results.

## 4. Conclusions

The CNTs functionalized with an APB-based DES were able to efficiently remove mercury ions from water solution. A comparative study of the NARX, LR and FFNN models was conducted based on their performance and accuracy; the NARX model presented better performance than the FFNN and LR models. The effect of various parameters, including adsorbent dosage, pH, contact time and mercury ion concentration, was investigated. Three kinetics models were applied on the predicted and experimental data such as intraparticle diffusion, pseudo first-order and pseudo second-order models, the pseudo second-order model described the data best. For the FFNN model, the maximum RE was 16.4%, *R*^2^ was 0.9313 and MSE was 2.27 × 10^−3^. The LR model had a maximum RE of 15.02%, an *R*^2^ of 0.9304 and an MSE of 2.2 × 10^−3^. The NARX model had a maximum RE of 9. 79%, an *R*^2^ of 0.9701 and an MSE of 1.15 × 10^−3^.

## Figures and Tables

**Table 1:** Adsorption kinetics and correlation coefficients.

			Pseudo First-Order ln(qe–qt) vs. Time (t)		Pseudo Second-Order (t/qt vs. t)		Intraparticle (qt vs. t^0.5^)	
Dose mg	pH	C0 mg/L	Experimental *R*^2^	NARX output *R*^2^	Experimental *R*^2^	NARX output *R*^2^	Experimental *R*^2^	NARX output *R*^2^
20	3	3	0.9378	0.9314	0.9978	0.9971	0.9114	0.9128
20	6	5	0.8689	0.8301	0.9959	0.9918	0.9053	0.9058
30	6	5	0.5692	0.5489	0.9969	0.9946	0.6045	0.6721

**Table 2:** Performance indicators.

Indicator	NARX	FFNN	LR
MSE	1.15 × 10^−3^	2.27 × 10^−3^	2.2 × 10^−3^
RMSE	3.40 × 10^−2^	4.81 × 10^−2^	4.74 × 10^−2^
RRMSE	6.24 × 10^−2^	7.92 × 10^−2^	7.61 × 10^−2^
MAPE	5.71%	6.92%	6.68%

## References

[1] Chen H.-R., Chen C.-C., Reddy A.S., Chen C.-Y., Li W.R., Tseng M.-J., Liu H.-T., Pan W., Maity J.P., Atla S.B. (2011). Removal of mercury by foam fractionation using surfactin, a biosurfactant. Int. J. Mol. Sci..

[2] Davodi B., Ghorbani M., Jahangiri M. (2017). Adsorption of mercury from aqueous solution on synthetic polydopamine nanocomposite based on magnetic nanoparticles using Box–Behnken design. J. Taiwan Inst. Chem. Eng..

[3] Khairi N., Yusof N., Abdullah A., Mohammad F. (2015). Removal of toxic mercury from petroleum oil by newly synthesized molecularly-imprinted polymer. Int. J. Mol. Sci..

[4] He C., Ren L., Zhu W., Xu Y., Qian X. (2015). Removal of mercury from aqueous solution using mesoporous silica nanoparticles modified with polyamide receptor. J. Colloid Interface Sci..

[5] Orr S., Bridges C. (2017). Chronic kidney disease and exposure to nephrotoxic metals. Int. J. Mol. Sci..

[6] Hsi H.-C., Lee H.-H., Hwang J.-F., Chen W. (2010). Mercury speciation and distribution in a 660-megawatt utility boiler in Taiwan firing bituminous coals. J. Air Waste Manag. Assoc..

[7] Das S.K., Das A.R., Guha A.K. (2007). A study on the adsorption mechanism of mercury on Aspergillus versicolor biomass. Environ. Sci. Technol..

[8] Jiang G.-B., Shi J.-B., Feng X.-B. (2006). Mercury pollution in China. Environ. Sci. Technol..

[9] Li S.-X., Feng-Ying Z., Yang H., Jian-Cong N. (2011). Thorough removal of inorganic and organic mercury from aqueous solutions by adsorption on Lemna minor powder. J. Hazard. Mater..

[10] Li B., Huang M., Fu T., Pan L., Yao W., Guo L. (2012). Microfiltration process by inorganic membranes for clarification of Tongbi liquor. Molecules.

[11] Chojnacki A., Chojnacka K., Hoffmann J., Gorecki H. (2004). The application of natural zeolites for mercury removal: From laboratory tests to industrial scale. Miner. Eng..

[12] Huttenloch P., Roehl K.E., Czurda K. (2003). Use of copper shavings to remove mercury from contaminated groundwater or wastewater by amalgamation. Environ. Sci. Technol..

[13] Biester H., Schuhmacher P., Müller G. (2000). Effectiveness of mossy tin filters to remove mercury from aqueous solution by Hg (II) reduction and Hg (0) amalgamation. Water Res..

[14] Oehmen A., Viegas R., Velizarov S., Reis M.A., Crespo J.G. (2006). Removal of heavy metals from drinking water supplies through the ion exchange membrane bioreactor. Desalination.

[15] Kyzas G., Deliyanni E. (2013). Mercury (II) removal with modified magnetic chitosan adsorbents. Molecules.

[16] Oubagaranadin J.U.K., Sathyamurthy N., Murthy Z. (2007). Evaluation of Fuller’s earth for the adsorption of mercury from aqueous solutions: A comparative study with activated carbon. J. Hazard. Mater..

[17] Lourie E., Gjengedal E. (2011). Metal sorption by peat and algae treated peat: Kinetics and factors affecting the process. Chemosphere.

[18] Azari A., Gharibi H., Kakavandi B., Ghanizadeh G., Javid A., Mahvi A.H., Sharafi K., Khosravia T. (2017). Magnetic adsorption separation process: An alternative method of mercury extracting from aqueous solution using modified chitosan coated Fe3O4 nanocomposites. J. Chem. Technol. Biotechnol..

[19] Davis A.P. (1993). Hazardous and industrial wastes. Proceedings of the Twenty-Fifth Mid-Atlantic Industrial Waste Conference, College Park, MD, USA, 7–9 July 1993.

[20] Keppert M., Doušová B., Reiterman P., Koloušek D., Záleská M., Černý R. (2018). Application of heavy metals sorbent as reactive component in cementitious composites. J. Clean. Prod..

[21] Abbas A., Al-Amer A.M., Laoui T., Al-Marri M.J., Nasser M.S., Khraisheh M., Atieh M.A. (2016). Heavy metal removal from aqueous solution by advanced carbon nanotubes: Critical review of adsorption applications. Sep. Purif. Technol..

[22] Tawabini B.S., Al-Khaldi S.F., Khaled M.M., Atieh M.A. (2011). Removal of arsenic from water by iron oxide nanoparticles impregnated on carbon nanotubes. J. Environ. Sci. Health Part A.

[23] Lu M., Ohba T., Kaneko K., Hata K., Yumura M., Iijima S., Komatsu H., Sakuma A., Kanoh H. (2013). Electron density modification of single wall carbon nanotubes (SWCNT) by liquid-phase molecular adsorption of hexaiodobenzene. Materials.

[24] Sun Y.-P., Fu K., Lin Y., Huang W. (2002). Functionalized carbon nanotubes: Properties and applications. Acc. Chem. Res..

[25] Fiyadh S.S., AlSaadi M.A., AlOmar M.K., Fayaed S.S., Mjalli F.S., El-Shafie A. (2018). BTPC-Based DES-Functionalized CNTs for A s 3+ Removal from Water: NARX Neural Network Approach. J. Environ. Eng..

[26] Garcia G., Atilhan M., Aparicio S. (2015). Interfacial properties of deep eutectic solvents regarding to CO2 capture. J. Phys. Chem. C.

[27] Garcia G., Aparicio S., Ullah R., Atilhan M. (2015). Deep eutectic solvents: Physicochemical properties and gas separation applications. Energy Fuels.

[28] Abbott A.P., Capper G., Davies D.L., Rasheed R.K., Tambyrajah V. (2003). Novel solvent properties of choline chloride/urea mixtures. Chem. Commun..

[29] Zhao B.-Y., Xu P., Yang F.-X., Wu H., Zong M.-H., Lou W.-Y. (2015). Biocompatible deep eutectic solvents based on choline chloride: Characterization and application to the extraction of rutin from Sophora japonica. Acs Sustain. Chem. Eng..

[30] Zhang Q., Vigier K.D.O., Royer S., Jérôme F. (2012). Deep eutectic solvents: Syntheses, properties and applications. Chem. Soc. Rev..

[31] Zhou C., Ding L., Skibniewski M.J., Luo H., Zhang H. (2018). Data based complex network modeling and analysis of shield tunneling performance in metro construction. Adv. Eng. Inform..

[32] Fiyadh S.S., AlSaadi M.A., AlOmar M.K., Fayaed S.S., Hama A.R., Bee S., El-Shafie A. (2017). The modelling of lead removal from water by deep eutectic solvents functionalized CNTs: Artificial neural network (ANN) approach. Water Sci. Technol..

[33] Fiyadh S.S., AlSaadi M.A., AlOmar M.K., Fayaed S.S., El-Shafie A. (2019). Lead removal from water using DES functionalized CNTs: ANN modeling approach. Desalin. Water Treat..

[34] Tanzifi M., Yaraki M.T., Kiadehi A.D., Hosseini S.H., Olazar M., Bharti A.K., Agarwal S., Gupta V.K., Kazemi A. (2018). Adsorption of Amido Black 10B from aqueous solution using polyaniline/SiO2 nanocomposite: Experimental investigation and artificial neural network modeling. J. Colloid Interface Sci..

[35] Fiyadh S.S., AlSaadi M.A., Jaafar W.Z.B., AlOmar M.K., Fayaed S.S., Hama A.R., Hin L.S., El-Shafie A. (2019). Mercury removal from water using deep eutectic solvents-functionalized multi walled carbon nanotubes: Nonlinear autoregressive network with an exogenous input neural network approach. Environ. Prog. Sustain..

[36] AlOmar M.K., Alsaadi M.A., Hayyan M., Akib S., Ibrahim M., Hashim M.A. (2017). Allyl triphenyl phosphonium bromide based DES-functionalized carbon nanotubes for the removal of mercury from water. Chemosphere.

[37] Kazemipour M., Ansari M., Tajrobehkar S., Majdzadeh M., Kermani H.R. (2008). Removal of lead, cadmium, zinc, and copper from industrial wastewater by carbon developed from walnut, hazelnut, almond, pistachio shell, and apricot stone. J. Hazard. Mater..

[38] Witek-Krowiak A., Szafran R.G., Modelski S. (2011). Biosorption of heavy metals from aqueous solutions onto peanut shell as a low-cost biosorbent. Desalination.

[39] Zabihi M., Asl A.H., Ahmadpour A. (2010). Studies on adsorption of mercury from aqueous solution on activated carbons prepared from walnut shell. J. Hazard. Mater..

[40] Bandaru N.M., Reta N., Dalal H., Ellis A.V., Shapter J., Voelcker N.H. (2013). Enhanced adsorption of mercury ions on thiol derivatized single wall carbon nanotubes. J. Hazard. Mater..

[41] Das B., Mondal N., Bhaumik R., Roy P. (2014). Insight into adsorption equilibrium, kinetics and thermodynamics of lead onto alluvial soil. Int. J. Environ. Sci. Technol..

[42] Zhang C., Sui J., Li J., Tang Y., Cai W. (2012). Efficient removal of heavy metal ions by thiol-functionalized superparamagnetic carbon nanotubes. Chem. Eng. J..

[43] AlOmar M.K., Hayyan M., Alsaadi M.A., Akib S., Hayyan A., Hashim M.A. (2016). Glycerol-based deep eutectic solvents: Physical properties. J. Mol. Liq..

[44] AlSaadi M.A., Al Mamun A., Alam M.Z., Amosa M.K., Atieh M.A. (2016). Removal of cadmium from water by CNT–PAC composite: Effect of functionalization. Nano.

[45] Wunsch A., Liesch T., Broda S. (2018). Forecasting groundwater levels using nonlinear autoregressive networks with exogenous input (NARX). J. Hydrol..

[46] Fayaed S.S., El-Shafie A., Jaafar O. (2013). Adaptive neuro-fuzzy inference system–based model for elevation–surface area–storage interrelationships. Neural Comput. Appl..

[47] Samarasinghe S. (2016). Neural Networks for Applied Sciences and Engineering: From Fundamentals to Complex Pattern Recognition.

[48] Govindaraju R.S. (2000). Artificial neural networks in hydrology. I: Preliminary concepts. J. Hydrol. Eng..

[49] Chen S., Billings S., Grant P. (1990). Non-linear system identification using neural networks. Int. J. Control.

[50] El-Shafie A.H., El-Manadely M.S. (2011). An integrated neural network stochastic dynamic programming model for optimizing the operation policy of Aswan High Dam. Hydrol. Res..

[51] El-Shafie A., Taha M.R., Noureldin A. (2007). A neuro-fuzzy model for inflow forecasting of the Nile river at Aswan high dam. Water Resour. Manag..

[52] Olaofe Z.O. (2014). A 5-day wind speed & power forecasts using a layer recurrent neural network (LRNN). Sustain. Energy Technol. Assess..

